# Modification and Validation of the Chinese Short-Form Aging Perception Questionnaire: A Psychometric Analysis

**DOI:** 10.3390/healthcare13131566

**Published:** 2025-06-30

**Authors:** Xinyi Liu, Wanhong Xiong, Dan Wang, Suting Song, Yu Luo

**Affiliations:** School of Nursing, Third Military University/Army Medical University, No. 30 Gaotanyan Street, Shapingba District, Chongqing 400038, China; xyliu7012@tmmu.edu.cn (X.L.); xwh516789412@tmmu.edu.cn (W.X.); wangdan0225@tmmu.edu.cn (D.W.); songst@tmmu.edu.cn (S.S.)

**Keywords:** community-dwelling older adults, self-perception of aging, psychometric analysis, classic test theory, item response theory

## Abstract

**Background/Objectives**: A reasonable assessment of the self-perception of aging (SPA) is of great significance to the health outcomes of older adults. This study aimed to develop the Modified Aging Perception Questionnaire (M-APQ) and to verify its psychometric properties. **Methods**: A multi-method study was conducted. In phase I, a qualitative study was conducted to supplement items to form the draft M-APQ. In phase II, three rounds of cognitive interviews were conducted to revise ambiguous items and form the prefinal M-APQ. In phase III, items were selected using Classical Test Theory (CTT) and Item Response Theory (IRT) to form the final M-APQ. In phase IV, the psychometric properties of the final version of M-APQ were validated. **Results**: Three items were added in Phase I. Six items were revised in Phase II. Eleven items were removed in phase III, leaving twenty-four items in the final version of M-APQ. In phase IV, the M-APQ showed good construct validity and convergent validity. The known-group validity analysis indicated significant differences in the M-APQ dimension scores on different self-rated health statuses. The Cronbach’s α for M-APQ and each dimension ranged from 0.798 to 0.888, and the intraclass correlation coefficients ranged from 0.704 to 0.883. The IRT analysis showed that item discrimination parameters ranged from 1.746 to 3.630, and difficulty parameters increased sequentially. **Conclusions**: The 24-item M-APQ includes seven dimensions and is a valid tool for assessing the self-perception of aging (SPA) among community-dwelling older adults.

## 1. Introduction

Globally, population aging is accelerating at an unprecedented pace. According to the World Population Prospects 2024 report, by the end of the 2070s, the number of people aged 65 and over is expected to surpass those under 18 [1]. At present, China’s aging population has a large scale and a rapid development speed. By the end of 2024, the total number of people aged 60 and over in China had exceeded 300 million, reaching 22.0% of the total population, and the size of the population aged 65 and over had exceeded 220 million, accounting for 15.6% of the total population [2].

As people age, they may face various physical, psychological, and social changes, such as functional decline, illness, increased loneliness, and social role changes [3,4]. The long-persisting negative stereotypes of older adults in society and the media, such as slow response and lack of energy, not only affect the attitudes of other groups towards older adults but also affect the subjective view of their aging [5]. Self-perception of aging (SPA) refers to the subjective cognitive and emotional responses formed by older adults under the influence of physiological, psychological, and social aging challenges [6]. The SPA is an important predictor of cognitive function [7], mental health [8], social relationships [9], and longevity [10].

The SPA is shaped by cultural context and prevailing social values [11]. In collectivist cultures, strong family and community ties are highly valued, and older adults are often seen as bearers of tradition and sources of wisdom [12]. In contrast, individualistic cultures tend to emphasize independence and productivity, which may inadvertently lead to the marginalization of older adults [13]. Research has shown that individuals in collectivist societies generally hold more positive attitudes toward older adults [14]. In the Chinese context, deeply rooted Confucian norms further influence the roles and expectations of older adults. Traditional values such as filial piety emphasize respect for and care for older adults, shaping their social status and perceptions of aging [15,16]. However, the rapid demographic aging is accompanied by increasing social pressures, and negative attitudes toward older adults have also begun to emerge within collectivist societies [17]. Moreover, accelerated socio-economic development, urbanization, and shifts in family structures have led to changes in intergenerational relationships and the gradual weakening of traditional support systems [18,19]. Collectivist values and expectations may also influence the internalization of age-related stereotypes regarding the roles and contributions of older adults within the family and society [20,21]. Given these factors, a culturally sensitive assessment of SPA is crucial for understanding and addressing the needs and well-being of Chinese community-dwelling older adults.

The validity of studies on SPA is highly dependent on the quality of its assessment tools. There are several tools available to assess SPA, such as the Ageing Cognitions Scales (Age-Cog) [22], the Aging Perception Questionnaire (APQ) [6], and the Age-Related Changes Questionnaire (AARC) [23]. However, researchers developed these instruments based on their research objectives or interpretations of SPA, and there are differences in measurement dimensions and theoretical basis. The research team previously systematically reviewed the psychometric properties of the existing assessment tools using the COSMIN method. The results showed that 10 of the 13 existing assessment tools were rated grade B, and three were rated grade C.

After discussion in the research group, it was found that the APQ has a high potential for application in the assessment of SPA. The original APQ, developed by Barker in 2007 based on the Common-Sense Model (CSM), comprises 49 items and eight dimensions: acute/chronic timeline, cyclical timeline, positive consequence, negative consequence, positive control, negative control, emotional representation, and identity [6]. In addition, the items included in the “identity” dimension aimed to assess the extent to which older adults attributed their health problems to “getting older.” Barker suggested that the remaining seven dimensions, except for the “identity” dimension, could be integrated into a scale, and the reliability and validity of this scale have been verified in different cultural contexts.

The Chinese version of the APQ (C-APQ), translated and adapted by Chen et al. [24], maintains the original multidimensional structure for use with Chinese older adults. Although its psychometric properties were validated in Shanghai community-dwelling older adults, C-APQ retains the length format of the original APQ, which may increase the response burden for older adults. Therefore, researchers in many countries have developed a short form of the APQ, effectively reducing the burden on participants while retaining the multidimensional structure of the original questionnaire.

Older adults from different cultures have some commonalities in their aging perception, but modernization processes, cultural differences, and social and family relationships may significantly affect their aging perception. In 2014, Sexton et al. developed the Brief APQ (B-APQ), a 17-item version based on data from the Irish Longitudinal Study of Ageing, which omitted the cyclical timeline dimension and combined negative control with negative consequences [25]. The B-APQ was later translated into Chinese by Hu and has been used in related studies [26]. However, combining dimensions in the B-APQ may introduce methodological bias and weaken the theoretical integrity. Recognizing this, Slotman et al. created the short version of the APQ (APQ-S), a 21-item form that retains all seven key dimensions of the original APQ, with demonstrated reliability and validity in the Dutch context [27]. This version has not yet been translated and adapted among older adults in China. Additionally, Miremadi et al. translated the original APQ into Persian and revised the questionnaire based on Iranian older adults in 2020, resulting in the Persian version of the APQ (APQ-P) containing 20 items and four dimensions [28].

In conclusion, the APQ, as an instrument developed based on the CSM, can comprehensively assess the multidimensional characteristics of older adults’ SPA. Although the theoretical structure of the original APQ is relatively complete, its large number of items may increase the fatigue of older adults when answering. While short forms like APQ-P and APQ-S offer improved convenience, they were developed using data from non-Chinese populations and have not been fully validated among Chinese community-dwelling older adults. Furthermore, the removal or combination of specific dimensions in existing short forms may compromise the theoretical comprehensiveness of SPA assessment.

Therefore, this study aimed to retain the core dimensions of the C-APQ and appropriately simplify items to reduce respondent burden, thereby developing the Chinese short-form APQ. The ultimate goal is to provide a scientific and reliable tool for assessing the SPA level of Chinese community-dwelling older adults and evaluating the effects of interventions. Healthcare professionals and community health workers can use the M-APQ for routine screening to identify older adults who may have negative perceptions of aging, allowing for early and targeted psychological or social interventions. Its brevity and ease of administration facilitate large-scale community-based health assessments and make it suitable for inclusion in annual health check-ups or home visits. In addition, the M-APQ can guide the design, implementation, and evaluation of evidence-based community health promotion programs to foster positive attitudes towards aging and ultimately improve health outcomes among older adults.

## 2. Materials and Methods

### 2.1. Design

**Figure 1:**
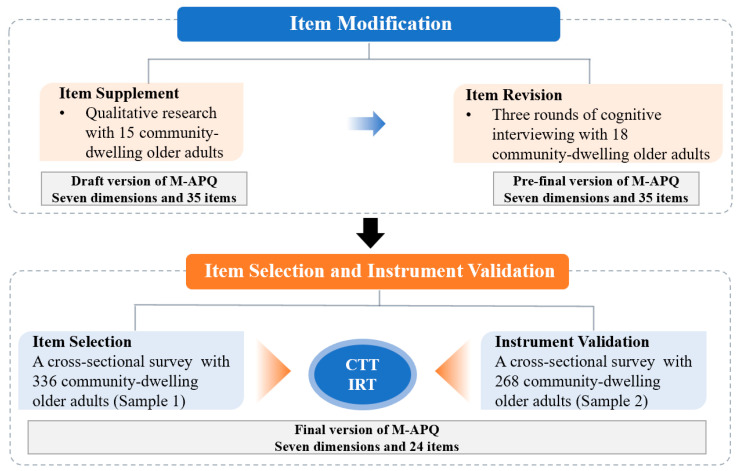
The modification process. M-APQ: Modified Aging Perception Questionnaire; CTT: Classic Test Theory; and IRT: Item Response Theory.

In this study, the C-APQ was revised through three stages: item supplement, item revision, and item selection to form the M-APQ and further verify its properties. The process is shown in Figure 1.

### 2.2. Item Modification

Phase I: Item Supplement Based on Qualitative Research.

The maximum variation of the purposive sampling method was used to select older adults aged 60 years old as research subjects from two communities in Chongqing [29]. Data were collected by one-to-one, face-to-face, and semi-structured in-depth interviews. The formal interview outline was formed based on CSM, including the following questions: (1) How do you think about “getting old”? (2) Do you feel that you are “getting old”? Why or why not? (3) What changes have you noticed in yourself as you age? (4) Do you think “getting old” is controllable? Why? (5) How do you think “getting old” has affected you? (6) What emotional responses do you have towards “getting old”? The interview ended with open-ended questions such as, “What do you think is important that we have not covered?” Or “Is there anything else you would like to add?” This study conducted formal interviews with 15 community-dwelling older adults, which reached data saturation [30]. Based on the interview results, a total of three items were added: Item 33, “As I get older, I feel more and more useless”; Item 34, “I am afraid of loneliness caused by growing old”; Item 35, “I worry about becoming a burden on others as I grow older.” These items consistently emerged as significant and culturally relevant themes from the qualitative data, reflecting common emotional responses to aging among Chinese community-dwelling older adults, particularly feelings of diminished value and fear of becoming a burden, which were not adequately captured by the original APQ items. On this basis, the draft M-APQ was formed.

Phase II: Item Revision Based on Cognitive Interviewing.

The maximum variation of the purposive sampling method was used to select older adults as research subjects from two communities in Chongqing. Cognitive interviewing was conducted by integrating the “think aloud” technique with “verbal probing” to refine the items in the draft M-APQ [31], and the pre-final version of M-APQ was formed. To ensure that the content of the interviews could comprehensively cover the questionnaire items, the research team referred to the Question Appraisal System and adjusted it appropriately in combination with the actual situation to develop the interview outline [32]. The research process was as follows: Participants completed the questionnaire, after which the researcher conducted in-depth interviews guided by the outline. After the cognitive interviewing was completed, the interview data were systematically organized and analyzed. In this phase, three rounds of interviews were conducted with 18 community-dwelling older adults: seven participants in the first round, six in the second, and five in the third. Throughout these interviews, a total of six items of the questionnaire were revised. After the revision of these items, the pre-final version of the M-APQ was formed.

### 2.3. Item Selection and Instrument Validation

Phase III: Item Selection Using CTT and IRT.

A convenience sampling method was used to recruit community-dwelling older adults from Chongqing for a questionnaire survey. The aim of Phase III is to select the items by combining IRT and CTT to form the final version of M-APQ.

Phase IV: Validation of Psychometric Properties of M-APQ.

A convenience sampling method was used to recruit community-dwelling older adults from Chongqing for a questionnaire survey. The aim of Phase IV is to validate the psychometric properties of M-APQ.

#### 2.3.1. Setting and Sample

The research subjects of this study were all community-dwelling older adults in Chongqing. Convenience sampling was employed to recruit participants from four teaching cooperative communities located in the main urban districts of Chongqing. Multiple strategies were utilized to promote the study, including posting advertisements in WeChat groups and Moments, distributing announcements and flyers in residential areas, and organizing large-scale publicity events in community centers and other public venues. Older adults interested in participating were invited to contact the research team or community staff for further information. Eligibility screenings were conducted by trained research group members through face-to-face or telephone interviews. The inclusion criteria were (a) age of 60 years or older; (b) residing in the Chongqing community; (c) possessing basic communication and comprehension skills; and (d) having been informed about and voluntarily participating in the study. The exclusion criteria were (a) communication barriers or an inability to express personal thoughts clearly and (b) withdrawal from the study for various reasons.

Sample sizes were determined by considering the requirements of IRT and CTT. According to the COMSIN guidelines, the optimal sample size for confirmatory factor analysis (CFA) should exceed seven times the number of items in the instrument and should not be fewer than 100 cases [33]. Furthermore, when calculating the required sample size, we estimated at least 10% of the participants to explain potentially invalid or incomplete questionnaires. Experts suggest that for the multi-parameter model of IRT, the sample size should be at least 200 cases to ensure accurate estimation of IRT parameters [34]. Considering all the above factors, the minimum sample size for Phase III was 272 cases, and that for Phase IV was 200 cases.

#### 2.3.2. Measurement

The measurement in Phase III included the general information questionnaire and the pre-final version of M-APQ. The measurement in Phase IV included the general information questionnaire, the final M-APQ, and the 12-item Short-form Health Survey (SF-12) [35].

The general information questionnaire included gender, age, education level, marital status, residence status, and self-assessed economic stress.

The pre-final version of M-APQ was formed by revising the items of the draft M-APQ based on cognitive interviewing. The final M-APQ was formed after item selection of the pre-final version of M-APQ by combining IRT and CTT.

The SF-12 is a tool simplified and developed by the Health Institute at the New England Medical Center(Boston, MA, USA.) based on the SF-36. This tool focuses on two dimensions: physical and mental health, which together provide a comprehensive overview of an individual’s overall health status. The physical health dimension, represented by the Physical Component Summary (PCS), includes four domains: physical functioning, role physical, bodily pain, and general health. The mental health dimension, represented by the Mental Component Summary (MCS), encompasses four domains as well: vitality, social functioning, role emotional, and mental health. The SF-12 serves as a practical, efficient, and reliable tool for assessing health-related quality of life of older adults in China.

#### 2.3.3. Data Collection

Data collection was conducted through questionnaire surveys. Before the questionnaire survey began, the researcher was required to obtain informed consent from the older adults and explain in detail how to fill out the questionnaire to those who met the inclusion and exclusion criteria. If older adults encountered any questions during the completion process, they could consult the researcher anytime. For individuals unable to complete the questionnaire independently, the researcher read each item and provided assistance in completing the questionnaire. After completing the questionnaire, the questionnaire was collected on the spot and checked by the researcher. If any omission is found, the questionnaire was supplemented promptly.

#### 2.3.4. Data Analysis

Statistical descriptions of the general information of community-dwelling older adults were performed. The item selection and validation of psychometric properties of M-APQ based on CTT were conducted using SPSS 27.0 and Amos 24.0 software. The item selection and validation of psychometric properties of M-APQ based on IRT were conducted using the Item package in R 4.4.1.

##### Item Selection

The item selection was conducted by combining CTT and IRT. An item was considered for deletion if it simultaneously met two or more specified exclusion criteria.

The CTT-based item selection methods included the following:

1. Frequency analysis: An item is considered for deletion if the selection rate for the highest or lowest response options exceeds 30%, indicating a potential ceiling or floor effect [36].
2. Coefficient of Variation (CV): The CV is calculated using the formula CV = Standard Deviation/Mean. An item is considered for deletion if its CV value is below 0.25 [37].
3. Correlation coefficient: An item is considered for deletion if it demonstrates a correlation coefficient of less than 0.4 with its belonged dimension or more than 0.4 with other dimensions [38].
4. Cronbach’s α coefficient: First, the Cronbach’s α coefficient for the whole M-APQ was calculated, and then each item was deleted one by one, and the change in Cronbach’s α coefficient for the remaining items was observed. If Cronbach’s α coefficient increases after deleting an item, the deletion of that item should be considered [39].
5. Factor analysis: Exploratory factor analysis (EFA) was adopted, and any item with less than 0.40 factor loading after rotation is considered for deletion [40].

The Graded Response Model is applicable to ordered multi-class data and was chosen for data analysis in this study [41]. The IRT-based item selection methods included the following:

1. Discrimination: The discrimination parameters can be classified into five grades: 0.01–0.34 (very low), 0.35–0.64 (low), 0.65–1.34 (medium), 1.35–1.69 (high), and ≥1.70 (very high). Items with very low discrimination should be considered for deletion [42].
2. Difficulty: For the Likert 5-level scale, each item should have four difficulty parameters, and it should satisfy *β*_1_ < *β*_2_ < *β*_3_ < *β*_4_. Additionally, the differences between these parameters should be less than five. If these conditions are not fulfilled, the item should be considered for deletion [43].
3. Item Characteristic Curves: For items with five response options, the ideal item characteristic curves should be presented as follows: The first curve is monotonically decreasing; the second, third, and fourth curves are approximately normally distributed; and the fifth curve is monotonically increasing. If these standards are not met, the item will be considered for deletion [43].

##### Instrument Validation

The validation of psychometric properties included assessments of feasibility, reliability, and validity, as well as an IRT-based assessment of the discrimination and difficulty of each item on the M-APQ and the item characteristic curves. The details are as follows:

1. Feasibility: The completion rate and completion time of the M-APQ were evaluated. The completion rate refers to the percentage of participants who completed the questionnaire, which is generally required to exceed 85% [44]. For older adults, the completion time should generally be less than 30 min [45].
2. Internal consistency: The internal consistency of the M-APQ and dimensions was measured by Cronbach’s α coefficient. When Cronbach’s α coefficient reaches 0.7, it indicates relatively good internal consistency [46].
3. Reliability: Two weeks after the first survey, a total of 30 community-dwelling older adults who participated in the first survey were selected for retesting. The Intraclass Correlation Coefficient (ICC) was calculated using a two-way random effects model. An ICC value less than 0.40 indicates low reliability, 0.40–0.75 indicates good reliability, and an ICC value greater than 0.75 reflects excellent reliability. If the 95% confidence interval does not include 0, it indicates that the reliability is significant [47].
4. Structural validity: The structural validity of M-APQ was evaluated by CFA, with the following fit indices employed: The chi-square degree of freedom ratio (*χ*^2^/*df*), where a ratio between 1 and 3 indicates a good model fit; the Root Mean Square Error of Approximation (RMSEA), where values less than 0.05 suggest excellent model fit, and values between 0.05 and 0.08 indicate an acceptable fit; the Root Mean Square Residual (RMR), in which lower values indicate better fit, and values less than 0.05 are generally preferred; and the Goodness of Fit Index (GFI), Normed Fit Index (NFI), Comparative Fit Index (CFI), Incremental Fit Index (IFI), and Tucker–Lewis Index (TLI), for which values greater than 0.90 are considered indicative of satisfactory model fit [48].
5. Convergent validity: Two approaches were used to assess convergent validity. First, the Average Variance Extracted (AVE) and Composite Reliability (CR) were calculated for each dimension. Convergent validity is acceptable when the AVE exceeds 0.5 and the CR exceeds 0.7 [49]. Second, the correlation coefficient between the M-APQ and the SF-12 was examined. A correlation coefficient of *r* < 0.30 indicates a weak correlation, 0.30 ≤ *r* < 0.60 indicates a moderate correlation, 0.60 ≤ *r* < 0.80 indicates a high correlation, and *r* ≥ 0.80 indicates a strong correlation [50]. Based on previous studies, it was hypothesized that the dimensions contained in the negative SPA of M-APQ are negatively correlated with the SF-12 dimension. In contrast, the dimensions contained in the positive SPA of M-APQ are positively correlated with the SF-12 dimension.
6. Known-group validity: It was hypothesized that there would be significant differences in M-APQ scores based on participants’ self-rated health status. Participants were categorized into two groups—“excellent/very good/good” and “fair/poor”—and the M-APQ scores were compared between these groups using the rank-sum test [51].

### 2.4. Ethical Considerations

This study was approved by the Medical Ethics Committee of the Army Medical University (2023 No. 9-01) and the Ethics Committee of Shuangbei Community Health Service Centre (No. SB202305-02). This study was conducted in accordance with the Declaration of Helsinki and the International Ethical Guidelines for Biomedical Research Involving Human Subjects. Before the commencement of the study, the researchers explained the purpose, procedures, and potential risks to the participants and ensured that they had sufficient time to understand the relevant information and decide whether or not to participate. Participants had the right to withdraw from the study at any stage without providing an explanation and without any adverse consequences. The privacy of all participants was protected, and all information collected was used solely for this study and was not disclosed to unauthorized parties.

## 3. Results

### 3.1. Characteristics of the Participants

A total of 336 community-dwelling older adults were surveyed in Phase III, and 268 were surveyed in Phase IV. The mean age of participants in Phase III was 69.32 years (SD = 6.31), while the mean age in Phase IV was 68.98 years (SD = 6.40). The general information of participants is shown in Table 1.

### 3.2. Phase III: Item Selection Using CTT and IRT

#### 3.2.1. Item Selection Based on CTT

1. Frequency analysis: The floor and ceiling effects of each item ranged from 1.19% to 22.69% and 15.82% to 29.25%, respectively (Table S1).
2. Coefficient of variation: The CV value for all items exceeded 0.25 (Table S1).
3. Correlation coefficient: The items with a correlation coefficient lower than 0.4 in their respective dimensions were Items 13, 15, 28, and 34 (Table S2).
4. Cronbach’s α coefficient: The overall Cronbach’s α coefficient of M-APQ was 0.739. The item-by-item deletion analysis found that the items with Cronbach’s α coefficients greater than 0.739 after deletion were Items 1, 2, 13, 14, 15, 16, 18, 23, 28, 30, and 34 (Table S1).
5. Factor analysis: Seven factors were extracted, accounting for a cumulative explained variance of 51.341%. The items with factor loading less than 0.4 included Items 1, 2, 13, 14, 15, 16, 18, 23, 28, 30, and 34 (Table S3).

The combined analysis found that Items 1, 2, 13, 14, 15, 16, 18, 23, 28, 30, and 34 met the criteria for deletion.

#### 3.2.2. Item Selection Based on IRT

The discrimination parameters of Items 3, 4, 5, 6, 7, 8, 9, 10, 11, 12, 17, 19, 20, 21, 22, 24, 25, 26, 27, 29, 31, 32, and 33 were at a medium or higher level, and the difficulty parameters of these items were within a reasonable range (Table S4). Meanwhile, the item characteristic curves of these items demonstrated relatively ideal distributions. Therefore, it is recommended that the above items be retained. The item characteristic curves of each item are shown in Figure S1.

Combining the results of CTT and IRT, in this study, Items 1, 2, 13, 14, 15, 16, 18, 23, 28, 30, and 34 were finally deleted, and 24 items were retained (Table 2).

### 3.3. Phase IV: Validation of the Psychometric Properties of M-APQ

**Figure 2:**
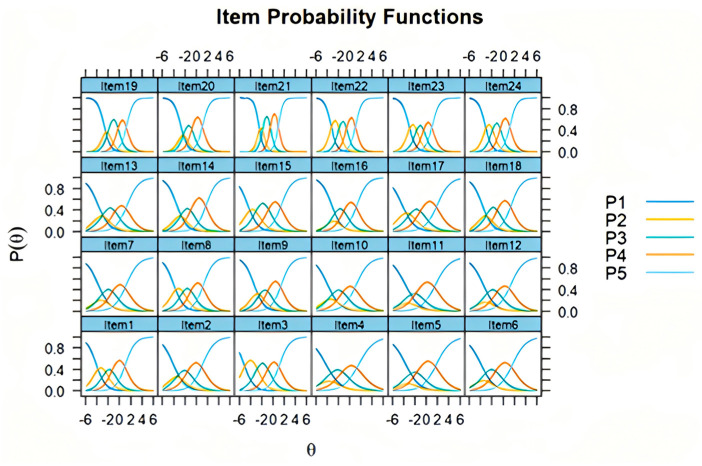
Item Characteristic Curves of items of M-APQ.

1. Feasibility: The research team checked the completion of the questionnaires during the survey, so the completion rate reached 100%. The time required to complete the questionnaire ranged from 10 to 19 min.
2. Internal consistency: The total Cronbach’s α coefficient of M-APQ was 0.882, and the Cronbach’s α coefficients of each dimension ranged from 0.798 to 0.888 (Table 3).
3. Reliability: The ICC values of each dimension of M-APQ were all greater than 0.70 (Table 3).
4. Structural validity: The results of CFA showed that all fit indices reached ideal levels (*χ*^2^/*df* = 1.346; RMSEA = 0.034; RMR = 0.037; GFI = 0.915; CFI = 0.977; NFI = 0.909; IFI = 0.977; TLI = 0.972).
5. Convergent validity: The AVE values for all dimensions were greater than 0.500, and the CR values were greater than 0.700 (Table 4). Spearman correlation analyses revealed that the acute/chronic timeline, cyclical timeline, negative consequence, and emotional representation dimensions of M-APQ negatively correlated with the physical and mental health dimensions of SF-12. The positive consequence, positive control, and negative control dimensions of M-APQ were positively correlated with the physical and mental health dimensions of SF-12 (Table 4).
6. Known-group validity: Each dimension of M-APQ showed significant differences among the community-dwelling older adults with different self-rated health statuses (Table 5).
7. Discrimination and difficulty: The IRT analysis results indicated that the discrimination parameters of each item ranged from 1.746 to 3.630. The difficulty parameters of each item gradually increased (Table 6). The item characteristic curves of each item are shown in Figure 2.

## 4. Discussion

According to the COSMIN guidelines, it is recommended to prioritize the selection and validation of existing instruments across diverse populations rather than developing new ones. Therefore, this study aims to optimize and revise the existing questionnaires. The optimization of instruments should begin with a clear conceptualization of the target construct to ensure comprehensive assessment [52]. Based on qualitative research on the SPA of community-dwelling older adults, three new items were added to the emotional representation dimension. Following analyses using both CTT and IRT, two of these items were retained.

Cognitive interviewing is an important part of instrument development and item optimization, aiming to ensure that the items and response options are relevant and clear to the target population [53]. Through cognitive interviewing, items or response options that are difficult to understand can be clarified, and words or terms that may be misunderstood can be identified, thereby enhancing the rigor of the instrument during the adaptation process [54]. Dickin et al. pointed out that even if the instrument has been translated into the native language, cognitive interviewing still needs to be conducted to ensure that the target population fully understands the items, especially for participants with lower educational levels [55]. For SPA instruments, it is crucial to accurately capture the real experiences of older adults. If the items do not adequately reflect their actual feelings, it may affect the structural validity and applicability of the instrument. Therefore, listening to the voices of older adults and ensuring that the items reflect their real-life experiences is an indispensable aspect of the questionnaire optimization. This study followed the COSMIN guidelines and conducted three rounds of cognitive interviewing to gain an in-depth understanding of how participants understood the items and the logic of their response selection [56]. Problems such as inappropriate wording, ambiguity, and redundancy were systematically addressed, aligning the instrument with the language habits and cultural context of community-dwelling older adults in China.

Currently, the C-APQ consists of 32 items, which can comprehensively assess the SPA of older adults in China and has been applied in relevant studies [24]. However, as a questionnaire with many items, it has limitations in practical applications, such as time consumption and inefficient response rates, so it is necessary to simplify it. Researchers have employed various methods to develop short versions of the original APQ. For example, Sexton et al. deleted the items by identifying overlapping pairs of items based on modified indices, parameter change values, and standardized residual covariance [25]. Miremadi et al. utilized EFA and CFA for item selection [28]. Slotman et al. conducted item selection by examining indicators such as local dependence, factor loading, floor effect, and ceiling effect [27]. In this study, CTT and IRT were employed to screen items. Ultimately, 11 items were removed, resulting in the M-APQ comprising seven dimensions and 24 items. Although this reduction is moderate compared to other short forms, the M-APQ retains all seven dimensions of the original scale. Specifically, except for the emotional representation dimension, which includes six items, each of the other six dimensions (acute/chronic timeline, cyclical timeline, negative control, positive control, negative consequence, and positive consequence) includes three items. This moderate reduction helps avoid excessive loss of important content, ensuring the instrument maintains its theoretical integrity and multidimensional structure.

In practical application, the M-APQ demonstrated good participant acceptability and feasibility. The items of the M-APQ were easy for the participants to understand, and the response rate was high. The time required to complete the M-APQ ranged from 10 to 19 min, which is reasonable and helps avoid interruptions. The number of items is appropriate, which enables comprehensive assessments of the target construct without increasing the excessive burden on participants, further proving the scientific validity and feasibility of the M-APQ. As a result, the M-APQ is easier to use in community settings, which may facilitate broader adoption and improve data quality.

The M-APQ shows good psychometric properties. The CFA results indicate that all fit indices for the seven-factor structure met established psychometric standards, suggesting that the M-APQ possesses a stable and well-defined factor structure. Compared with the B-APQ (five dimensions) [57] and APQ-P (four dimensions) [28], the M-APQ includes seven dimensions. This multidimensional structure aligns closely with the theoretical framework of the Common-Sense Model and the original APQ, ensuring that the instrument comprehensively reflects the complex and multifaceted nature of SPA. The results of known-group validity showed significant differences in the scores of each dimension of M-APQ between community-dwelling older adults with different self-rated health statuses. This finding is consistent with theoretical expectations and previous research, showing that older adults with better self-rated health are more likely to possess positive aging perceptions [58]. In contrast, those with poorer health or multiple chronic conditions are more prone to negative SPA [59]. These results provide strong evidence for the known-group validity of the M-APQ, confirming its effectiveness in differentiating between population subgroups with varying health statuses.

Convergent validity was assessed by calculating the AVE and CR for each dimension of the M-APQ. The AVE values for all dimensions ranged from 0.572 to 0.685, exceeding the commonly accepted threshold of 0.50 [49], while the CR values ranged from 0.800 to 0.889, surpassing the recommended minimum of 0.70 [60]. These results provide strong evidence that the items within each dimension of M-APQ share a substantial proportion of common variance, indicating that they consistently measure the same underlying construct. Additionally, the results of the Spearman correlation analyses between SPA and health-related quality of life were consistent with theoretical expectations and previous research findings [25]. Specifically, older adults who exhibited a stronger awareness of the chronic or cyclical nature of their aging tended to report lower levels of mental health. Conversely, those who perceived aging as having a positive impact on their lives reported higher levels of mental health, supporting the notion that a positive outlook on aging can enhance mental well-being. Furthermore, a greater sense of control over the positive aspects of aging was correlated with better physical health, indicating that perceived agency in managing the aging process may contribute to improved physical health status [61]. The alignment between observed correlations and existing research findings demonstrates that the M-APQ meaningfully relates to important health outcomes. These findings further reinforce its convergent validity.

Cronbach’s α coefficient is a commonly used indicator for evaluating internal consistency. In this study, all dimensions of the M-APQ demonstrated Cronbach’s α values exceeding 0.70, indicating that both the overall scale and its subdimensions possess acceptable internal consistency. Compared to previous studies, the internal consistency of the M-APQ and its dimensions in this study is relatively good. For example, in the APQ-S, Cronbach’s α for the “cyclical timeline” dimension in the Turkish version [61] and the “positive control” dimension in the Dutch version [27] were slightly below the generally accepted threshold, at 0.60 and 0.53, respectively. In the B-APQ, the “emotional representation” dimension in the Malaysian version [62] and the “positive consequences” dimension in the Persian version [63] both had α values below 0.70. These variations may be attributed to differences in item content, cultural adaptation, or the number of items within individual dimensions. Therefore, our findings indicate that the M-APQ demonstrates good homogeneity among its items.

The reliability of the M-APQ was further examined using ICC, with all dimensions demonstrating values exceeding 0.700. This result indicates a strong test–retest reliability, suggesting that the scale yields stable and consistent scores when administered at different time points. High ICC values are significant for longitudinal research or repeated assessments, as they confirm that the instrument is not unduly influenced by temporal fluctuations or random error [47]. Such stability is critical for ensuring that the observed scores reflect actual changes in the self-perceptions of aging rather than measurement error. This level of reliability enhances confidence in the M-APQ’s capacity to accurately monitor changes or stability in aging perceptions over time, supporting its suitability for both research and clinical applications.

The results of the IRT analysis indicated that the discrimination parameters for all M-APQ items were at a medium or higher level, suggesting that each item can effectively differentiate respondents with varying levels of the latent trait. High discrimination values are important, as they reflect the ability of each item to distinguish subtle differences in SPA among individuals. Moreover, the difficulty parameters for each item exhibited an orderly and increasing progression across response options, with differences between response categories not exceeding the commonly accepted threshold of 5 points—a standard considered acceptable in IRT applications [64]. This finding indicates that the response categories form a well-graded continuum, allowing the scale to capture a broad spectrum of attitudes and perceptions. The distribution of item characteristic curves was also found to be ideal, demonstrating that each item performs consistently and as expected across different latent trait levels. The original APQ and its short forms in various languages have not been evaluated using IRT. IRT provides unique insights into item functioning, such as differential item functioning and measurement precision, which are challenging to uncover with traditional psychometric approaches [65]. Overall, these IRT findings provide strong evidence for the measurement precision and validity of the M-APQ, further supporting its psychometric soundness for use in both research and applied settings.

Cultural values have an important influence on shaping the SPA of older adults. The newly added item 33 in the “emotional representation” dimension of M-APQ, “As I grow older, I feel increasingly useless,” captures the self-perceived sense of uselessness. This item reflects an individual’s negative self-evaluation of their own and others’ usefulness (such as family, friends, community, or society) and also reflects their overall understanding and acceptance of the aging process [66]. This cognitive tendency is shaped by personal experiences and the daily living environment [67]. In Confucian culture, older adults are usually regarded as core family and society members, attaching importance to family and intergenerational relationships, which helps them maintain a positive self-image [68]. However, as modernization and industrialization progress, the traditional views of filial piety have somewhat diminished. Older persons’ status in family decision-making and social roles may be challenged, and their sense of purpose may be weakened accordingly. Some older adults may internalize these negative views and develop a sense of uselessness [69]. To provide a comprehensive assessment of older adults’ SPA, instruments need to be adapted to specific cultural and linguistic contexts. For example, during the validation of the APQ short form for older persons with chronic disease, the expert panel suggested that items related to a “sense of uselessness” should be added according to cultural characteristics [70]. Additionally, Zhao et al. proposed that self-perceived uselessness is an important aspect of SPA [71]. This view can be traced back to the relevant item in the Philadelphia Geriatric Center Morale Scale, which has been used to assess SPA in longitudinal studies [72].

Furthermore, item 35 was added to the emotional representation dimension of M-APQ: “I am worried about becoming a burden to my family due to getting old.” The original expression of this item was “I am worried about becoming a burden to others due to aging.” Participants suggested limiting “others” to “family members” in cognitive interviewing. Qualitative research shows that older adults generally mention that “children have their own little families” and express that they “do not want to cause trouble.” They believe that adult children have taken on work and family responsibilities and should not impose additional burdens by taking care of their parents [73]. Subsequent research found that when older adults require short-term care, they generally hope for visits from their children, but they do not wish their children to adjust their work and family arrangements due to long-term care for them [74]. As individuals age, they are more likely to experience declines in their ability to live independently due to health issues or financial challenges, leading to a sense of “being a burden,” especially when they need more family care and support [75].

### 4.1. Implications for Healthcare

This study developed a convenient and scientific instrument to assist healthcare professionals and researchers in effectively assessing the SPA level of community-dwelling older adults. Compared with C-APQ, M-APQ also covers seven dimensions and comprehensively reflects the SPA status of older adults. Meanwhile, the number of items is concise, which improves the convenience and efficiency of use.

In practice, the M-APQ can serve as a screening tool during routine geriatric health assessments, enabling healthcare providers to identify older adults with negative SPA and potential risk for adverse physical and mental health outcomes. By analyzing scores across specific dimensions, healthcare providers can pinpoint areas where individuals may have cognitive biases or require additional support and develop tailored intervention programs. For example, older adults with low scores in the “positive control” dimension can be referred to participate in emotional regulation training, problem-solving skills workshops, or programs that strengthen social support networks, thereby improving their capacity to cope positively with aging. For those with high scores in the “Emotional Representation” dimension, priority can be given to psychological counseling, group psychotherapy, or art therapy interventions to alleviate negative emotions and foster a more positive outlook on aging.

At the policy and programmatic level, regular assessment and aggregation of SPA data among community older adults allow administrators to monitor population-level trends in SPA. This evidence can guide the optimal allocation of public health resources and inform the design and implementation of targeted health promotion and mental health support initiatives, ultimately advancing healthy aging goals.

### 4.2. Strengths and Limitations

The strength of this study lies in its systematic and rigorous optimization process of the instrument. Through qualitative research exploring the concept connotation of SPA among community-dwelling older adults, three new items were added to the C-APQ. Following three rounds of cognitive interviewing, the items were revised based on participant feedback to address potential comprehension differences, ensuring that the semantics of the items remained consistent with the original APQ. The items were screened using a combination of CTT and IRT, ultimately resulting in the formation of the M-APQ, and its psychometric properties were further verified.

However, this study has some limitations. The data for the questionnaire survey were collected in the Chongqing area, and participants were recruited through convenience sampling, which may limit the generalizability of the results. To determine the applicability of these findings to other regions, further validation through multi-center studies and larger sample sizes is needed. Additionally, not all psychometric properties mentioned in the COSMIN guidelines were assessed. Future studies should evaluate the criterion validity of M-APQ by comparing it with C-APQ and adopt longitudinal study designs to assess its responsiveness to further verify the scientific nature of M-APQ.

## 5. Conclusions

This study optimized C-APQ through item supplementation, revision, and screening to form M-APQ, providing an instrument for evaluating the SPA of community-dwelling older adults. M-APQ consists of seven dimensions and 24 items, conforms to the language expression habits and cultural context of China, and demonstrates high feasibility as well as good reliability, validity, and discrimination. It provides a scientific and feasible instrument for evaluating the SPA status and intervention effects in community-dwelling older adults.

## Figures and Tables

**Table 1:** Characteristics of the participants.

Variable	Sample 1 (n = 336)		Sample 2 (n = 268)	
	Number (n)	Proportion (%)	Number (n)	Proportion (%)
Gender				
Male	134	39.88	113	42.16
Female	202	60.12	255	57.84
Age (Year)				
60~69	198	58.93	166	61.94
70~79	115	34.23	80	29.85
80~	23	6.84	22	8.21
Education level				
Primary school or below	95	28.27	86	32.09
Junior high school	150	44.64	131	48.88
High school	76	22.62	40	14.93
College or above	15	4.46	11	4.10
Employment status				
Unemployed	321	95.54	249	92.91
Employed	15	4.46	19	7.09
Marital status				
Married	277	82.44	222	82.84
Divorced	13	3.87	17	6.34
Widowed	46	13.69	29	10.82
Living arrangement				
Living with spouse	172	51.19	113	42.16
Living with children	58	17.26	54	20.15
Living with spouse and children	73	21.73	67	25.00
Living alone	24	7.14	16	5.97
Other	9	2.68	18	6.72
Perceived financial pressure				
No pressure	186	55.36	159	59.33
Some pressure	115	34.23	90	33.58
Significant pressure	35	10.41	19	7.09
Chronic disease status				
No	123	36.61	111	41.42
Yes	213	63.39	157	58.58
Self-rated health				
Excellent	25	7.44	24	8.96
Very good	87	25.89	89	33.21
Good	69	20.54	62	23.13
Fair	122	36.31	83	30.97
Poor	33	9.82	10	3.73

**Table 2:** Results of item selection based on CTT and IRT.

Item	Text	Retain/Delete
Item 1	I am conscious of getting older all of the time.	delete
Item 2	I am always aware of my age.	delete
Item 3	I always classify myself as old.	retain
Item 4	I am always aware of the fact that I am getting older.	retain
Item 5	I feel my age in everything that I do.	retain
Item 6	As I get older I get wiser.	retain
Item 7	As I get older I continue to grow as a person.	retain
Item 8	As I get older I appreciate things more.	retain
Item 9	I get depressed when I think about how ageing might affect the things that I can do.	retain
Item 10	The quality of my social life in later years depends on me.	retain
Item 11	The quality of my relationships with others in later life depends on me.	retain
Item 12	Whether I continue living life to the full depends on me.	retain
Item 13	I get depressed when I think about the effect that getting older might have on my social life.	delete
Item 14	As I get older there is much I can do to maintain my independence.	delete
Item 15	Whether getting older has positive sides to it depends on me.	delete
Item 16	Getting older restricts the things that I can do.	delete
Item 17	Getting older makes me less independent.	retain
Item 18	Getting older makes everything a lot harder for me.	delete
Item 19	As I get older I can take part in fewer activities.	retain
Item 20	As I get older I do not cope as well with problems that arise.	retain
Item 21	Slowing down with age is not something I can control.	retain
Item 22	How mobile I am in later life is not up to me.	retain
Item 23	I have no control over whether I lose vitality or zest for life as I age.	delete
Item 24	I have no control over the effects which getting older has on my social life.	retain
Item 25	I get depressed when I think about getting older.	retain
Item 26	I worry about the effects that getting older may have on my relationships with others.	retain
Item 27	I go through cycles in which my experience of ageing gets better and worse.	retain
Item 28	My awareness of getting older comes and goes in cycles.	delete
Item 29	I feel angry when I think about getting older.	retain
Item 30	I go through phases of feeling old.	delete
Item 31	My awareness of getting older changes a great deal from day to day.	retain
Item 32	I go through phases of viewing myself as being old.	retain
Item 33	As I get older, I feel more and more useless.	retain
Item 34	I am afraid of loneliness caused by growing old.	delete
Item 35	I worry about becoming a burden on others as I grow older.	retain

**Table 3:** Results of internal consistency and reliability.

Dimension	Cronbach’s α Coefficient	ICC (95% *CI*)
Acute/chronic timeline	0.807	0.883 (0.770~0.943)
Cyclical timeline	0.863	0.729 (0.504~0.861)
Negative control	0.866	0.769 (0.569~0.883)
Positive control	0.865	0.717 (0.486~0.855)
Negative consequence	0.798	0.704 (0.466~0.847)
Positive consequence	0.822	0.742 (0.526~0.868)
Emotional representation	0.888	0.720 (0.490~0.856)

**Table 4:** Results of convergent validity.

Dimension	AVE	CR	Correlation Between Dimensions of SF-12	
			Physical Health	Mental Health
Acute/chronic timeline	0.591	0.812	−0.237 ***	−0.266 ***
Cyclical timeline	0.682	0.865	−0.323 ***	−0.362 ***
Negative control	0.685	0.867	0.293 ***	0.313 ***
Positive control	0.684	0.866	0.305 ***	0.249 ***
Negative consequence	0.572	0.800	−0.372 ***	−0.304 ***
Positive consequence	0.608	0.823	0.252 ***	0.259 ***
Emotional representation	0.573	0.889	−0.391 ***	−0.345 ***

Note. *** *p* < 0.001.

**Table 5:** Results of known-group validity.

Dimension	Excellent/Very Good/Good (n = 156)	Fair/Poor (n = 112)	*Z*
Acute/chronic timeline	4.00 (3.67, 4.00)	4.33 (4.00, 4.67)	−5.302 ***
Cyclical timeline	3.67 (3.33, 4.00)	4.33 (3.67, 4.67)	−5.573 ***
Negative control	4.00 (3.67, 4.59)	3.67 (3.00, 4.00)	−4.956 ***
Positive control	4.00 (3.67, 4.67)	3.67 (3.00, 4.00)	−4.443 ***
Negative consequence	3.67 (3.00, 4.00)	4.00 (3.67, 4.33)	−4.830 ***
Positive consequence	4.00 (3.67, 4.33)	3.67 (3.00, 4.00)	−5.043 ***
Emotional representation	3.83 (3.17, 4.00)	4.17 (3.83, 4.50)	−5.538 ***

Note. *** *p* < 0.001.

**Table 6:** Discrimination and difficulty parameters of items.

Item	Discrimination α	Difficulty β_1_	Difficulty β_2_	Difficulty β_3_	Difficulty β_4_
Item 1	2.852	−2.725	−1.703	−0.668	0.720
Item 2	2.910	−2.358	−1.677	−0.687	0.638
Item 3	1.746	−3.820	−2.208	−0.631	0.947
Item 4	2.319	−2.204	−1.700	−0.502	0.826
Item 5	3.630	−1.887	−1.493	−0.522	0.858
Item 6	2.767	−2.157	−1.642	−0.487	0.896
Item 7	2.351	−2.284	−1.696	−0.561	0.765
Item 8	3.537	−2.477	−1.516	−0.422	0.817
Item 9	3.135	−2.136	−1.367	−0.388	0.959
Item 10	3.294	−2.072	−1.500	−0.467	0.645
Item 11	3.070	−1.899	−1.510	−0.613	0.797
Item 12	2.599	−2.003	−1.585	−0.455	0.787
Item 13	2.176	−2.462	−1.648	−0.392	0.910
Item 14	2.039	−2.521	−1.752	−0.508	1.296
Item 15	2.878	−2.772	−1.879	−0.451	0.952
Item 16	2.063	−2.349	−1.788	−0.572	0.939
Item 17	2.399	−2.568	−1.611	−0.448	1.107
Item 18	2.753	−2.466	−1.739	−0.537	0.944
Item 19	2.142	−2.709	−1.720	−0.153	1.180
Item 20	2.402	−2.564	−1.743	−0.505	0.979
Item 21	3.452	−2.657	−1.748	−0.331	0.887
Item 22	2.135	−3.287	−1.896	−0.439	1.006
Item 23	1.828	−3.083	−1.661	−0.338	1.022
Item 24	2.231	−3.017	−1.727	−0.358	1.092

## Data Availability

The data presented in this study are available on request from the corresponding author due to the privacy of participants.

## Supplementary Materials

The following supporting information can be downloaded at https://www.mdpi.com/article/10.3390/healthcare13131566/s1. Table S1: Results of frequency analysis, CV, and Cronbach’s α coefficient; Table S2: Item–dimension correlations: respective dimension and other dimensions; Table S3: Results of explanatory factor analysis; Table S4: Discrimination and difficulty parameters of items; Table S5: The final version of M-APQ; Figure S1: Item Characteristic Curves of items of pre-final M-APQ.

## Abbreviations

The following abbreviations are used in this manuscript:

SPA	Self-perception of Aging
EFA	Exploratory Factor Analysis
CFA	Confirmatory Factor Analysis
IRT	Item Response Theory
CTT	Classical Test Theory
APQ	Aging Perception Questionnaire
M-APQ	Modified Aging Perception Questionnaire
