# Supplementary material for: Modification and Validation of the Chinese Short-Form Aging Perception Questionnaire: A Psychometric Analysis
Source: Healthcare (Basel). 2025 Jun 30;13(13):1566. doi: 10.3390/healthcare13131566 (PMC12249300; doi:10.3390/healthcare13131566)
Supplement: Supplementary file 1 [file healthcare-13-01566-s001.zip › healthcare-3689321-supplementary.pdf]

Supporting Information

Table S1 Results of frequency analysis, CV and Cronbach's α coefficient

Item	Frequency analysis		CV	Cronbach's α coefficient
	Strongly disagree	Strongly agree		
Item1	0.218	0.206	0.482	0.753
Item2	0.209	0.173	0.476	0.750
Item3	0.027	0.254	0.251	0.725
Item4	0.045	0.287	0.268	0.727
Item5	0.018	0.254	0.242	0.727
Item6	0.042	0.248	0.259	0.728
Item7	0.027	0.182	0.244	0.729
Item8	0.030	0.212	0.238	0.728
Item9	0.012	0.212	0.229	0.723
Item10	0.045	0.272	0.268	0.729
Item11	0.060	0.245	0.271	0.728
Item12	0.060	0.263	0.281	0.727
Item13	0.185	0.191	0.459	0.747
Item14	0.221	0.173	0.483	0.743
Item15	0.197	0.239	0.477	0.755
Item16	0.212	0.218	0.479	0.748
Item17	0.048	0.293	0.273	0.728
Item18	0.188	0.164	0.464	0.741
Item19	0.030	0.230	0.259	0.727
Item20	0.048	0.221	0.278	0.727
Item21	0.036	0.260	0.270	0.728
Item22	0.042	0.158	0.256	0.727
Item23	0.227	0.194	0.485	0.750
Item24	0.015	0.203	0.227	0.730
Item25	0.033	0.182	0.257	0.721
Item26	0.024	0.212	0.257	0.721
Item27	0.048	0.236	0.265	0.731
Item28	0.203	0.203	0.468	0.746
Item29	0.036	0.212	0.246	0.722
Item30	0.215	0.209	0.475	0.746
Item31	0.051	0.218	0.260	0.729
Item32	0.042	0.248	0.257	0.730
Item33	0.024	0.203	0.235	0.720
Item34	0.185	0.251	0.461	0.755
Item35	0.021	0.197	0.245	0.725

Note. CV: Coefficient of Variation

Table S2. Item-dimension correlations: respective dimension and other dimensions

Dimension	Item	Acute/chronic timeline	Positive consequence	Positive control	Negative control	Negative consequence	Emotional representation	Cyclical timeline
Acute/chronic timeline	Item1	0.494	-0.083	0.026	-0.004	0.030	-0.131	-0.070
	Item2	0.458	-0.004	0.018	0.014	-0.032	-0.029	-0.040
	Item3	0.702	0.188	0.113	0.119	0.210	0.304	0.183
	Item4	0.721	0.187	0.137	0.108	0.091	0.224	0.196
	Item5	0.659	0.212	0.152	0.08	0.105	0.278	0.204
Positive consequence	Item6	0.145	0.857	0.078	0.155	0.196	0.188	0.183
	Item7	0.093	0.847	0.096	0.104	0.263	0.201	0.161
	Item8	0.098	0.857	0.097	0.185	0.220	0.250	0.126
Positive control	Item10	0.154	0.109	0.703	0.117	0.050	0.206	0.131
	Item11	0.111	0.100	0.706	0.112	0.043	0.281	0.131
	Item12	0.121	0.153	0.716	0.183	0.083	0.212	0.118
	Item14	0.070	0.049	0.476	0.059	-0.030	0.041	0.108
	Item15	-0.034	-0.063	0.331	-0.020	-0.064	0.034	-0.079
Negative control	Item21	0.100	0.290	0.074	0.769	0.129	0.191	0.196
	Item22	0.135	0.284	-0.003	0.728	0.139	0.272	0.205
	Item23	0.035	-0.053	-0.071	0.552	-0.022	0.042	-0.040
	Item24	0.059	0.308	0.082	0.681	0.141	0.194	0.165
Negative consequence	Item16	-0.046	0.036	0.017	0.042	0.466	0.013	0.003
	Item17	0.078	0.183	0.149	0.096	0.717	0.196	0.117
	Item18	0.120	-0.036	0.095	-0.029	0.411	0.081	0.151
	Item19	0.061	0.219	0.104	0.183	0.738	0.232	0.143

	Item20	0.081	0.183	0.084	0.119	0.719	0.279	0.147
Emotional representation	Item25	0.123	0.244	0.288	0.227	0.160	0.749	0.185
	Item29	0.194	0.211	0.207	0.151	0.196	0.740	0.164
	Item33	0.184	0.272	0.255	0.222	0.227	0.753	0.141
	Item9	0.156	0.238	0.183	0.232	0.213	0.695	0.141
	Item13	-0.035	-0.001	-0.030	0.034	0.020	0.307	0.088
	Item26	0.168	0.255	0.238	0.211	0.236	0.705	0.118
	Item34	-0.047	-0.101	-0.059	-0.003	0.005	0.212	0.011
	Item35	0.138	0.212	0.232	0.173	0.149	0.681	0.127
Cyclical timeline	Item27	0.140	0.135	0.075	0.092	0.154	0.122	0.674
	Item28	0.016	0.040	0.048	0.123	0.031	0.051	0.353
	Item30	0.010	0.065	0.046	0.041	0.042	0.038	0.458
	Item31	0.104	0.135	0.156	0.122	0.127	0.190	0.689
	Item32	0.109	0.171	0.048	0.132	0.154	0.204	0.682

Table S3. Results of explanatory factor analysis

Item	Rotated Factor Loadings						
	1	2	3	4	5	6	7
Item1	-0.160	-0.037	0.059	-0.124	-0.175	0.321	0.056
Item2	-0.030	0.028	-0.021	-0.064	0.006	0.198	-0.147
Item3	0.242	0.024	0.042	0.068	0.056	0.807	0.145
Item4	0.165	0.075	0.087	0.083	0.097	0.828	-0.004
Item5	0.208	0.020	0.098	0.122	0.136	0.742	0.004
Item6	0.111	0.105	0.024	0.067	0.801	0.112	0.113
Item7	0.123	0.047	0.045	0.012	0.800	0.054	0.188
Item8	0.223	0.076	0.042	0.014	0.815	0.041	0.123
Item9	0.719	0.182	0.116	0.072	0.079	0.058	0.093
Item10	0.203	0.007	0.858	-0.019	0.018	0.071	0.008
Item11	0.224	0.053	0.858	0.006	0.003	0.000	-0.006
Item12	0.170	0.141	0.826	-0.025	0.059	0.033	0.008
Item13	0.055	-0.038	-0.048	0.135	-0.009	-0.040	-0.032
Item14	0.035	0.012	0.108	0.141	0.105	0.091	-0.177
Item15	0.123	-0.059	-0.248	-0.015	-0.072	-0.074	-0.140
Item16	-0.014	0.022	0.003	-0.011	0.051	-0.038	0.018
Item17	0.131	0.868	0.058	-0.006	0.074	0.041	0.042
Item18	0.127	-0.026	0.001	0.080	-0.064	0.126	-0.232
Item19	0.126	0.870	0.054	0.020	0.090	0.003	0.135
Item20	0.204	0.860	0.048	0.041	0.050	0.034	0.063
Item21	0.117	0.071	0.129	0.067	0.140	0.063	0.801
Item22	0.217	0.069	-0.021	0.131	0.106	0.107	0.769
Item23	0.092	-0.204	-0.134	-0.109	-0.172	0.029	0.230
Item24	0.128	0.155	0.079	0.085	0.192	0.027	0.747
Item25	0.770	0.106	0.097	0.163	0.091	0.036	-0.002
Item26	0.745	0.071	0.080	0.025	0.090	0.072	0.103
Item27	0.000	0.069	0.115	0.804	0.046	0.159	0.152
Item28	0.005	0.183	0.054	-0.238	0.010	0.092	0.041
Item29	0.804	-0.001	0.062	0.050	0.035	0.105	0.021
Item30	-0.023	-0.108	0.129	0.047	0.123	0.049	-0.010
Item31	0.105	0.082	0.094	0.866	0.044	0.065	0.070
Item32	0.110	0.094	0.014	0.844	0.082	0.066	0.059
Item33	0.808	0.111	0.080	0.043	0.107	0.100	0.098
Item34	-0.092	0.065	-0.136	-0.063	-0.182	-0.004	0.101
Item35	0.766	0.037	0.059	0.055	0.073	0.078	0.027

Table S4. Discrimination and difficulty parameters of items

Item	Discrimination α	Difficulty β_1	Difficulty β_2	Difficulty β_3	Difficulty β_4
Item1	-0.176	7.326	2.256	-1.759	-7.733
Item2	-0.063	21.256	5.488	-7.683	-24.973
Item3	1.050	-4.022	-2.545	-1.165	1.203
Item4	0.868	-3.994	-2.888	-1.304	1.214
Item5	0.963	-4.786	-3.03	-1.000	1.293
Item6	0.976	-3.682	-2.881	-0.970	1.337
Item7	0.941	-4.327	-2.875	-0.911	1.86
Item8	1.098	-3.723	-2.805	-1.005	1.439
Item9	1.841	-3.506	-2.002	-0.635	1.064
Item10	0.850	-4.066	-3.134	-1.096	1.286
Item11	0.822	-3.797	-3.125	-1.395	1.548
Item12	0.874	-3.596	-2.961	-1.002	1.331
Item13	0.042	-35.711	-11.286	11.263	34.749
Item14	0.154	-8.217	-2.076	2.895	10.194
Item15	-0.138	10.224	2.970	-2.294	-8.403
Item16	0.005	-247.365	-83.427	71.699	240.688
Item17	0.884	-3.853	-2.946	-1.132	1.141
Item18	0.049	-29.985	-6.819	11.123	33.310
Item19	1.001	-4.008	-2.533	-0.963	1.407
Item20	1.058	-3.365	-2.334	-0.810	1.417
Item21	0.971	-3.897	-2.640	-0.774	1.287
Item22	1.098	-3.381	-2.477	-0.822	1.804
Item23	-0.062	19.889	6.360	-6.207	-23.154
Item24	1.016	-4.695	-3.060	-0.885	1.580
Item25	1.788	-2.718	-1.892	-0.378	1.203
Item26	1.560	-3.253	-1.921	-0.546	1.139
Item27	0.710	-4.567	-3.510	-1.331	1.810
Item28	0.082	-16.745	-5.727	3.177	16.770
Item29	1.639	-2.796	-2.094	-0.778	1.117
Item30	0.039	-32.828	-12.489	8.716	33.745
Item31	0.807	-4.030	-3.220	-1.379	1.749
Item32	0.821	-4.240	-3.377	-1.136	1.513
Item33	2.334	-2.628	-1.921	-0.526	0.992
Item34	-0.209	7.154	2.621	-0.788	-5.278
Item35	1.569	-3.374	-2.095	-0.544	1.207

Table S5. The final version of M-APQ

Item<br>Chinese (English)	Strongly disagree	Disagree	Neither agree nor disagree	Agree	Strongly agree
1.我总把自己归为老年人。					
（1.I always classify myself as old）					
2.我总是意识到自己在变老的事实。					
（2.I am always aware of the fact that I am getting older）					
3.我在做每件事情时，都能感觉到自己的年龄。					
（3.I feel my age in everything that I do）					
4.随着年龄的增长，我越来越明智。					
（4.As I get older I get wiser）					
5.随着年龄的增长，我仍在成长。					
（5.As I get older I continue to grow as a person）					
6.随着年龄的增长，我更懂得珍惜。					
（6.As I get older I appreciate things more）					
7.晚年的社交生活质量取决于我自己。					
（7.The quality of my social life in later years depends on me）					
8.晚年的人际关系质量取决于我自己。					
（8.The quality of my relationships with others in later life depends on me）					
9.是否能继续充实地生活取决于我自己。					
（9.Whether I continue living life to the full depends on me）					
10.变老会使我的依赖性越来越大。					
（10.Getting older makes me less independent）					
11.随着变老，我能参加的活动越来越少。					
（11.As I get older I can take part in fewer activities）					
12.随着变老，我不能很好地处理遇到的问题。					

（12.As I get older I do not cope as well with problems that arise）
13.因年龄增长而导致的行动迟缓是我无法控制的事情。
（13.Slowing down with age is not something I can control）
14.晚年是否行动不便不取决于我自己。
（14.How mobile I am in later life is not up to me）
15.变老对社交生活的影响是我无法控制的。
（15.I have no control over the effects which getting older has on my social life）
16.一想到自己在变老，我就感到难过。
（16.I get depressed when I think about getting older）
17.一想到自己在变老，我就感到生气。
（17.I feel angry when I think about getting older）
18.随着年龄的增长，我觉得自己越来越无用了。
（18.As I get older, I feel more and more useless）
19.一想到变老会影响我现在能做的事情，我就感到难过。
（19.I get depressed when I think about how ageing might affect the things that I can do）
20.我担心变老会影响人际关系。
（20.I worry about the effects that getting older may have on my relationships with others）
21.我担心因变老成为家人的负担。
（21.I worry about becoming a burden on family members as I grow older）
22.我对变老的感觉时好时坏。
（22.I go through cycles in which my experience of ageing gets better and worse）
23.我对“衰老”的认识和看法每天都会有变化。
（23.My awareness of getting older changes a great deal from day to day）
24.我对自己老年人身份的接受经历了不同的阶段。
（24.I go through phases of viewing myself as being old）

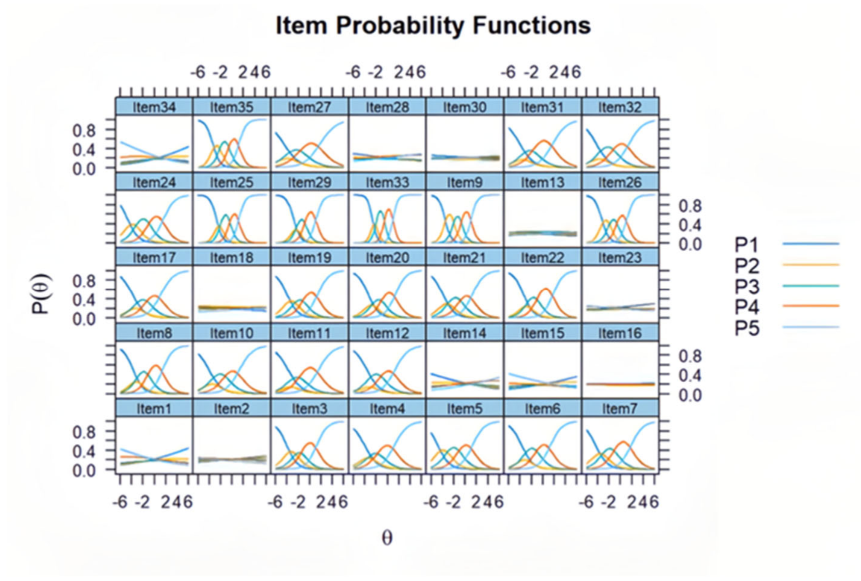

Figure S1. Item Characteristic Curves of items of pre-final M-APQ
